# Global effect of RpoS on gene expression in pathogenic *Escherichia coli *O157:H7 strain EDL933

**DOI:** 10.1186/1471-2164-10-349

**Published:** 2009-08-03

**Authors:** Tao Dong, Herb E Schellhorn

**Affiliations:** 1 Department of Biology Life Sciences Building, Rm. 433, McMaster University, 1280 Main Street, West Hamilton, ON L8S 4K1, Canada

## Abstract

**Background:**

RpoS is a conserved stress regulator that plays a critical role in survival under stress conditions in *Escherichia coli *and other γ-proteobacteria. RpoS is also involved in virulence of many pathogens including *Salmonella *and *Vibrio *species. Though well characterized in non-pathogenic *E. coli *K12 strains, the effect of RpoS on transcriptome expression has not been examined in pathogenic isolates. *E*. *coli *O157:H7 is a serious human enteropathogen, possessing a genome 20% larger than that of *E. coli *K12, and many of the additional genes are required for virulence. The genomic difference may result in substantial changes in RpoS-regulated gene expression. To test this, we compared the transcriptional profile of wild type and *rpoS *mutants of the *E. coli *O157:H7 EDL933 type strain.

**Results:**

The *rpoS *mutation had a pronounced effect on gene expression in stationary phase, and more than 1,000 genes were differentially expressed (twofold, P < 0.05). By contrast, we found 11 genes expressed differently in exponential phase. Western blot analysis revealed that, as expected, RpoS level was low in exponential phase and substantially increased in stationary phase. The defect in *rpoS *resulted in impaired expression of genes responsible for stress response (e.g., *gadA*, *katE *and *osmY*), arginine degradation (*astCADBE*), putrescine degradation (*puuABCD*), fatty acid oxidation (*fadBA *and *fadE*), and virulence (*ler*, *espI *and *cesF*). For EDL933-specific genes on O-islands, we found 50 genes expressed higher in wild type EDL933 and 49 genes expressed higher in the *rpoS *mutants. The protein levels of Tir and EspA, two LEE-encoded virulence factors, were elevated in the *rpoS *mutants under LEE induction conditions.

**Conclusion:**

Our results show that RpoS has a profound effect on global gene expression in the pathogenic strain O157:H7 EDL933, and the identified RpoS regulon, including many EDL933-specific genes, differs substantially from that of laboratory K12 strains.

## Background

Enterohemorrhagic *Escherichia coli *O157:H7 is a serious human pathogen that is responsible for many food-borne epidemic outbreaks, and the infection of *E. coli *O157:H7 can cause bloody diarrhea, hemorrhagic colitis and the hemolytic uremic syndrome [1,2]. The pathogenesis caused by *E. coli *O157:H7 is a complex process that requires a coordinated expression of virulence factors and regulators [1]. Known virulence factors in *E. coli *include the type III secretion factors encoded on the LEE pathogenicity island [3] and Shiga toxins (StxI and StxII) (reviewed in [4]). Many regulators are involved in mediating expression of these virulence factors. For example, genes on the LEE island are under control of H-NS [5], IHF [5], ClpXP [6] and three LEE-encoded regulators Ler, GrlA, and GrlR [7].

In *E. coli *and many other gamma-proteobacteria, the global stress response is controlled by the stationary phase sigma factor RpoS [8,9]. RpoS is induced in many stress conditions, including near-UV exposure [10], acid shock [11], heat shock [12], oxidative stress [10], and starvation [13], many of which *E. coli *may experience during growth and survival in natural environments. RpoS controls a large regulon consisting of 10% of the genome in *E. coli *K12 strains in stationary phase and stress conditions [14-17]. Even in exponential phase when RpoS is expressed at low levels, mutation in *rpoS *affects the expression of a large set of genes as well [18,19], and RpoS is important for DNA damage response in early exponential phase cells [20]. Though there is an identifiable core set of RpoS-regulated genes, the RpoS-dependence of many genes within the RpoS regulon varies depending on experimental conditions and strain backgrounds [16,18,19].

The effect of RpoS on virulence has been examined in many pathogens, and results differ depending on species. RpoS is critical for virulence of *Salmonella *[21] and *Vibrio cholerae *[22]. By contrast, RpoS does not appear to be required for virulence in *P. aeruginosa *[23] and *Y. enterocolitica *[24]. How RpoS is involved in enteropathogenesis of *E. coli *remains elusive, primarily because of the lack of a proper animal model since mice are not susceptible to infection of *E. coli *pathogens [25]. To overcome this problem, a model of using *Citrobacter rodentium*, a natural mouse enteropathogen closely related to *E. coli *has been widely used to simulate *E. coli *infection [25]. We have found that RpoS is important for full virulence of *C. rodentium *[26], suggesting an important role of RpoS in *E. coli *infection. Consistently, there are a few virulence traits regulated by RpoS. For example, curli production, important for virulence of *Salmonella *and *E. coli*, is positively regulated by RpoS [26-29]. The effect of RpoS on expression of the LEE virulence genes appears to vary depending on strain backgrounds and experimental conditions. For example, Sperandio et al. (1999) reported that the LEE3 operon and *tir *are positively regulated by RpoS in EHEC strain 86-24 [30]. However, in EHEC O157:H7 Sakai strain, LEE expression is enhanced in *rpoS *mutants [6,31]. It is likely that the expression of LEE genes is modulated differently depending on strain backgrounds. Surprisingly, expression of LEE genes appears to differ between O157:H7 Sakai and EDL933 strains as well (see Fig. 1 in [32]). The role of RpoS in strain EDL933 has not been tested. Furthermore, there has been no genomic profiling specifically investigating the involvement of RpoS in regulation of virulence genes in enteropathogenic *E. coli *and other related pathogens.

The genomes of *E. coli *K12 reference strain MG1655 and O157:H7 strain EDL933 differ considerably [33]. EDL933 and MG1655 possess 5.5 Mb and 4.6 Mb genome sizes, respectively, sharing 4.1 Mb backbone DNA [33]. DNA segments that are unique to one or the other strain and scattered within each genome are termed "O-islands" in O157:H7 and "K-islands" in K12 [33]. O-islands consist of 1.34 Mb DNA sequence encoding 26% of all EDL933 genes, while K-islands consist of 0.53 Mb harboring 12% of the genes in MG1655 genome [33]. Many genes on the O-islands are important in pathogenicity (e.g., genes on the LEE islands) [33]. In addition, gene polymorphisms on the backbone are common, since 75% of the backbone genes encode proteins that differ by at least one amino acid in these two strains [33]. Some genes are extremely divergent. In the case of *yadC*, the protein sequence in K12 and O157:H7 is only 34% identical [33]. The genome divergence between O157:H7 and K12 may have a substantial effect on gene regulation.

*E. coli *O157:H7 diverged from K12 strain about 4.5 million years ago [34], and genes on O-islands have been acquired through horizontal gene transfer [33-35]. How O-island genes are integrated into preexisting regulatory circuits controlled by RpoS is still unknown. Given that RpoS is known to regulate genes of nonessential functions [8,9,15,16], it is possible these O-island genes are preferentially under control of RpoS rather than RpoD, the housekeeping sigma factor. This has yet to be tested.

To examine RpoS-regulated gene expression in a pathogenic strain, we employed the *E. coli *O157:H7 strain EDL933 since this strain can cause serious human health problems and its genome is fully sequenced [33]. To compare with our previous results [15,18], we sampled wild type and isogenic *rpoS *mutants of EDL933 under the same growth conditions and compared their transcriptome expression in exponential phase (OD_600 _= 0.3) and early stationary phase (OD_600 _= 1.5). Herein we report that *rpoS *mutation had a profound effect on transcriptome expression. Genes under control of RpoS included many EDL933-specific genes on the O-islands. Besides stress response genes, RpoS also regulated the expression of genes involved in metabolic pathways, transcription, and virulence.

## Results

### Expression of RpoS during growth in LB media

**Figure 1:**
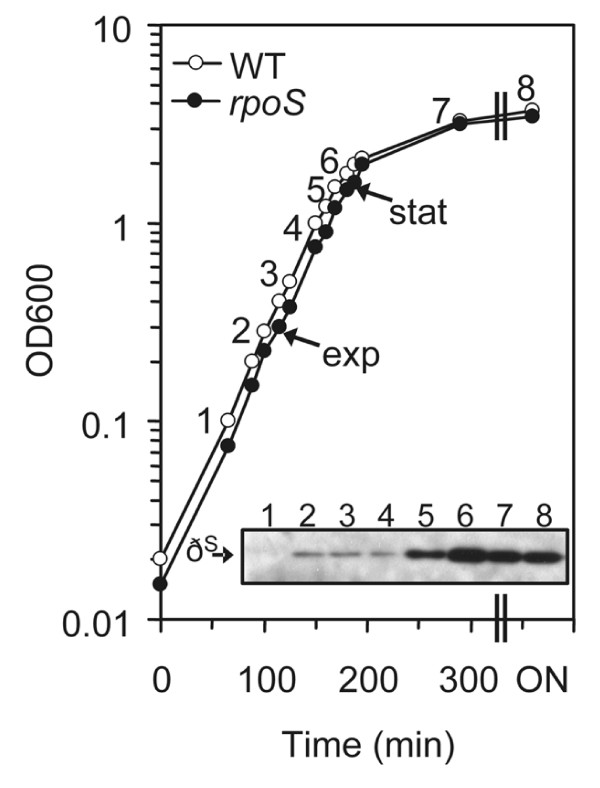
**Growth of EDL933 in LB media**. Cultures were inoculated from overnight cultures to a starting OD_600 _= 0.0001 and incubated aerobically at 37°C at 200 rpm. RNA samples were isolated at OD_600 _= 0.3 and 1.5 as indicated. RpoS (ð^S^) protein levels were tested by Western blot analyses using monoclonal anti-RpoS antiserum as described in Materials and Methods. This experiment was performed in triplicate using independent isolates. Averaged values were used for construction of the growth curve.

Although RpoS controls the expression of a large set of genes, mutation of *rpoS *has little effect on growth rate of *E. coli *K12 strain MG1655 [17,18]. To test whether this is applicable to pathogenic *E. coli *EDL933, we compared the growth of *rpoS *mutants with wild type EDL933 grown in LB. Both the growth rate and the time to enter stationary phase were similar between wild type and *rpoS *mutants of EDL933 (Figure 1). The generation time in exponential phase was approximately 26 min. This equivalence is important for comparison of genomic expression since the expression of many genes is affected by growth rate [36]. As expected, the protein level of RpoS was found to be low in early exponential phase, followed by a substantial increase during entry of stationary phase (Figure 1).

### Expression of genes under control of RpoS

**Figure 2:**
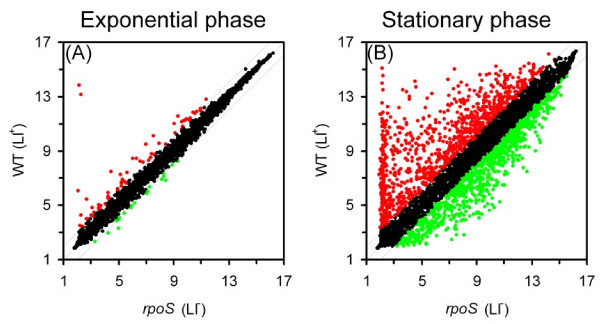
**Transcriptome profile of WT EDL933 and *rpoS *mutants**. Scatterplot was used to examine the effects of RpoS on gene expression in exponential (A) and stationary (B) phase. Probe sets (including genes and intergenic regions) are outlined by two parallel lines into three different groups: probe sets expressed at least twofold higher in the WT (red), those expressed more than twofold higher in *rpoS *mutants (green), and those not differentially expressed (black). LI: log_2_-transformed expression intensity.

The mutation in *rpoS *had a pronounced effect on genomic expression of EDL933 in stationary phase but a minor effect in exponential phase (Figure 2). In exponential phase when RpoS protein level was low, we found that 11 genes were differentially expressed in the *rpoS *mutants (Table 1), while in stationary phase, more than 1,000 genes were expressed differently as a result of *rpoS *mutation (twofold, P < 0.05) (Table 2 and Additional file 1). The false discovery rate was 1.4%. Among these stationary phase genes, 596 genes were expressed higher in the wild type EDL933, including 105 previously known RpoS-dependent genes in K12 strains. In addition, a mutation in *rpoS *led to increased expression of 536 genes (Table 3 and Additional file 1), indicating that the negative effect of RpoS on gene expression is also extensive. For genes on O-islands that are specific to EDL933, 50 genes showed higher expression in wild type and the expression of 49 genes was elevated in the *rpoS *mutants.

**Table 1:** RpoS-dependent genes in exponential phase (MER ≥ 2, P < 0.05).

Gene	RpoS-dependence (MER)	Function	Major regulator
motAB*	5/6	Flagellar motor complex	RpoF CpxR
*yciF*	6	Putative structural protein	H-NS
*yhjH*	8	Protein involved in flagellar function	RpoF FlhDC
Z1344	2	Putative endonuclease	
Z2774	3	Unknown	
Z3023	2	Putative secreted protein	
Z3024	4	Unknown	
Z3026	2	Putative secreted protein	
Z3672	4	Unknown	
Z4850	2	Putative O-methyltransferase	

* Indicates that some genes in the known operon are not listed because these genes did not satisfy the criteria to be RpoS-dependent.

**Table 2:** Top 100 most RpoS-dependent genes in stationary phase.

Gene	RpoS-dependence (MER)	Function	Major regulator
abgABT*	24/41/26	Aminoacyl aminohydrolase family proteins/transporter	AbgR
*aceBA*	164/422	Glyoxylate cycle	IclR FruR IHF CRP ArcA
*acs-yjcH-actP*	541/357/163	Acetyl-CoA synthetase/Unknown/Acetate permease	Fis IHF CRP
*aidB*	79	Isovaleryl CoA dehydrogenase	RpoS Ada Lrp
*puuCB*	576/214	Putrescine degradation II	
*astCADBE*	3492/1270/2402/512/388	Arginine degradation	RpoS RpoN ArgR NtrC
*blc*	568	Outer membrane lipoprotein	RpoS
csiD-ygaF-gabD*	357/67/44	Carbon starvation-induced gene/L-2-hydroxyglutarate oxidase/succinate semialdehyde dehydrogenase	RpoS CRP HNS CsiR Lrp
*csiE*	792	Stationary phase inducible protein	RpoS CRP HNS
*cstA*	46	Peptide transporter	CRP
*ddpXA*	39/31	D-ala-D-ala dipeptidase/transporter	RpoN NtrC
dppABDF*	74/64/148/122	Dipeptide ABC transporter	FNR IHF PhoB
*ecnB*	67	Entericidin B	RpoS
*espI*	78	Virulence protein	
*fadBA*	26/125	Fatty acid β-oxidation I	Fis ArcA FadR
*fadE*	74	Fatty acid β-oxidation I	FadR ArcA
*fadH*	64	2,4-dienoyl-CoA reductase	
fadI*	77	Fatty acid β-oxidation I	FadR ArcA
*fucAO*	32/123	Fucose catabolic process	FucR CRP
*gadAXW*	66/46/2	Glutamate dependent acid resistance	RpoS Fis FNR GadEXW CRP H-NS TorR
*galS*	140	GalS transcriptional dual regulator	GalS GalR CRP
*garD*	41	Galactarate dehydratase	CdaR
garPLR*	40/56/21	Degradation of D-glucarate and D-galactarate	H-NS FNR CadR
*hcaR*	46	Transcriptional activator of hca cluster	HcaR ArcA
*katE*	416	Catalase HPII	RpoS Fis
lsrABF*	46/118/124	Putative ABC transporter	RpoS CRP LsrR
*lsrR*	46	LsrR transcriptional repressor	CRP LsrR
*malKLM*	40/5/6	Maltose transport	RpoS MalT CRP
msyB*	40	Acidic protein	RpoS
*osmY*	27	Osmotically inducible protein	RpoS IHF CRP Fis
*otsBA*	211/220	Trehalose biosynthesis I	RpoS
*phnB*	56	Unknown	
potFGH*	52/18/4	Putrescine ABC transporter	RpoN NtrC
*poxB*	787	Pyruvate oxidase	
*prpR*	416	DNA-binding transcriptional activator	PrpR RpoN CRP
*psiF*	73	Phosphate starvation-induced protein	
*puuA*	393	Putrescine degradation II	
sufABCDS*	124/88/71/43/25	Fe-S cluster assembly	OxyR IHF IscR Fur RpoS
*talA*	67	Transaldolase A	RpoS
*tam*	86	Trans-aconitate methyltransferase	RpoS
*tdcBCD*	41/5/5	Threonine degradation I	
*tktB*	168	Transketolase II	RpoS
*tnaLAB*	443/189/750	Tryptophan catabolism	RpoS CRP TorR
*treF*	45	Cytoplasmic trehalase	
*ugpBAECQ*	161/129/46/184/4	Glycerol-3-P ABC transporter	PhoB CRP
*xylFGHR*	265/7/10/5	Xylose ABC transporter	RpoS Fis CRP XylR
*yahO*	241	Unknown	RpoS
*ybaST*	19/70	Glutaminase/ABC transporter	GadX RpoS
*ybgS*	82	Unknown	RpoS
*ybhPO*	251/7	Predicted DNase/cardiolipin synthase	RpoS
*ycaC*	653	Predicted hydrolase	BaeR Fnr RpoS
*ycaP*	66	Unknown	
*ycgB*	478	Unknown	RpoS
*yciGFE*	205/405/38	Unknown	RpoS HNS
*ydbC*	100	Predicted oxidoreductase	
ydcST*	125/22	Putative ABC transporter	RpoS
*yeaGH*	771/458	Protein kinase/Unknown	RpoS RpoN NtrC
*yeaT*	106	Unknown	
*yeaX*	48	Predicted oxidoreductase	
*yebV*	72	Unknown	
*yedI*	60	Unknown	
*yedK*	43	Unknown	
*yedK*	43	Unknown	
*yegP*	185	Unknown	RpoS
*yegS*	112	Lipid kinase	
yehZYX*	787/95/60	ABC transporter	RpoS RpoH
*yeiCN*	64/31	Unknown	
*yfcG*	187	Glutathione S-transferase	
*ygaM*	155	Stress-induced protein	RpoS
*ygdI*	90	Unknown	
*ygeV*	55	Putative transcriptional regulator	
*yghA*	326	Unknown	
*yhbO*	231	Stress response protein	RpoS
*yhcO*	214	Unknown	RpoS
*yhfG-fic*	133/111	Unknown/Stationary phase protein	RpoS
*yhjD*	41	Unknown	
*yhjY*	55	Putative lipase	
*yiaG*	449	Predicted transcriptional regulator	RpoS
*yjfN*	43	Unknown	
*yjgB*	55	Putative oxidoreductase	
*yjjM*	70	Predicted transcriptional regulator	
*ykgC*	127	Predicted oxidoreductase	
*yncB*	57	Predicted oxidoreductase	
*yniA*	63	Unknown	
*yodD*	290	Unknown	
*yphA*	135	Inner membrane protein	
*ytfQRT-yjfF*	879/76/36/34	Putative ABC transporter	
Z0608	55	Putative outer membrane protein	
*Z1504*	93	Unknown	
Z1629	117	Unknown	
Z1923	64	Prophage CP-933X protein	
Z1924	137	Prophage CP-933X protein	
Z2296	57	Unknown	
Z2297	254	Unknown	
Z2298	55	Unknown	
Z3624	64	D-fructokinase	
Z3625	139	Sucrose hydrolase	
Z4874	60	Unknown	
Z5000	48	Putative regulatory protein	
Z5352	125	Unknown	

* Indicates that some genes in the known operon are not listed because these genes did not satisfy the criteria to be RpoS-dependent.

**Table 3:** Top 50 RpoS-negatively regulated genes in stationary phase. MER: mean expression ratio (*rpoS*/WT).

Gene	MER	Function	Major regulator
*ampG*	-13	Muropeptide Major facilitator superfamily (MFS) transporter	
*ansP*	-12	L-asparagine permease	
ccmBC*	-8/-24	Protoheme IX ABC transporter	
*cmr*	-9	MFS multidrug transporter	
*codBA*	-26/-5	Cytosine transporter/deaminase	Nac PurR
*dusC*	-13	tRNA dihydrouridine synthase	
*emrAB*	-4/-11	EmrAB-TolC multidrug efflux	MprA
*endA*	-9	DNA-specific endonuclease I	
*guaBA*	-16/-6	Purine nucleotides *de novo *biosynthesis I	Fis CRP PurR DnaA
*lpxT*	-14	Und-PP pyrophosphatase	
*mscK*	-9	Mechanosensitive (MS) channel	
*napFD*	-13/-4	Ferredoxin-type protein/chaperone for NapA	NarL NarP FNR FlhDC ModE
*ndh*	-12	NADH dehydrogenase II	Fis FNR ArcA PdhR IHF
*pdhR*	-10	Pyruvate dehydrogenase regulator	CRP FNR PdhR
*proVWX*	-10/-6/-2	Proline ABC transporter	H-NS
*purEK*	-22/-18	Purine nucleotides de novo biosynthesis I	PurR
*purT*	-27	Purine nucleotides *de novo *biosynthesis I	
*pyrD*	-21	Dihydroorotate oxidase	PurR Fis
*pyrL*	-39	Pyr operon leader peptide	
*rarD*	-9	Putative permease	
*rhlE*	-18	ATP-dependent RNA helicase	
*rsxABCDGE-nth*	-10/-4/-7/-13/-26/-7/-16	SoxR reducing system/endonuclease III	
*speC*	-10	Putrescine biosynthesis III	CRP
*thiI*	-12	Thiamine biosynthesis	
*tyrP*	-15	Tyrosine transporter	TyrR
*uhpABC*	-5/-9/-18	Uptake of hexose phosphates	
*uraA*	-13	Uracil transport	
*xseA*	-10	Exonuclease VII	CRP
*yaaH*	-11	Inner membrane protein	
*yccFS*	-36/-27	Inner membrane protein	
*ychM*	-27	Unknown function	
*ydeA*	-35	MFS transporter	
*ydeP*	-12	Acid resistance protein	EvgA
*yegD*	-14	Actin family protein	
*ygiR*	-12	Unknown function	
*yhfC*	-40	MFS transporter	ArcA
*yhhQ*	-15	Unknown function	
*yhjV*	-14	Putative transporter protein	
*yieG*	-17	Putative transporter protein	
*yliG*	-14	Unknown function	
*ynjE*	-22	Putative sulfur transferase	
*yoaG*	-28	Unknown function	
*yobD*	-28	Unknown function	
Z2059	-11	Prophage CP-933O protein	
Z2274	-20	Unknown function	
Z2389	-9	Prophage CP-933R protein	
Z2605	-20	Putative arginine/ornithine antiporter	
Z2751	-15	Unknown function	
Z3622	-9	Putative resolvase	
Z4223	-13	Unknown function	

* Indicates that some genes in the known operon are not listed because these genes did not satisfy the criteria to be RpoS-dependent.

- Indicates negative regulation.

### RpoS-regulated functions in exponential phase

The expression of 11 genes was impaired in *rpoS *mutants in exponential phase (Table 1). Three genes, *motAB *and *yhjH*, are involved in the motor function of flagella. The gene *yciF*, encoding a putative structural protein, is RpoS-dependent in K12 strains [16]. There were seven EDL933-specific unknown genes under control of RpoS, two of which, Z3023 and Z3026, encode putative secreted proteins and play a role in colonization of *E. coli *O157:H7 in the bovine GI tract [37]. By contrast, the *rpoS *mutation had a much larger impact on gene expression in stationary phase. We thus focused on the analysis of the RpoS regulon in stationary phase.

### RpoS-regulated functions in stationary phase

#### Stress response

**Figure 3:**
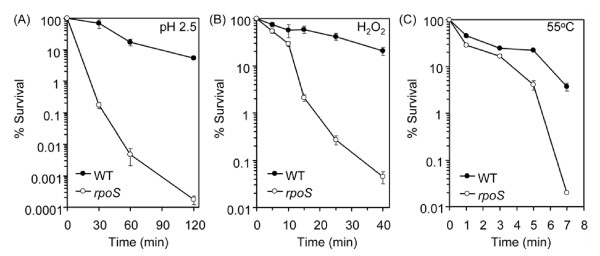
**Effect of *rpoS *mutation on survival under stress**. Stationary phase cultures were washed and diluted in 0.9% NaCl before exposure to low pH (2.5) (A), H_2_O_2 _(15 mM) (B), and heat (55°C) (C). WT, wild type EDL933; *rpoS*, *rpoS *mutant.

As expected, many of the identified RpoS up-regulated genes were those that are important for stress response. For example, the *rpoS *mutation resulted in decreased expression of stress response genes *yhiO *(*uspB*), *yhbO, gadAXW, gadB, gadE, osmY, csiD*, and *katE *that are known be RpoS-dependent in K12 strains [38]. The genes *gadAXW*, *gadB*, and *gadE *are important for acid resistance [39], *osmY *for hyperosmotic resistance [40], *yhiO *(*uspB*) for ethanol tolerance [41], *katE *for oxidative response [42,43], and *yhbO *for survival under oxidative, heat, UV, and pH stresses [16,44]. Consistently, survival of *rpoS *mutants under low pH, oxidative stress, and heat exposure was severely impaired in comparison with wild type EDL933 strain (Figure 3).

Two starvation-induced genes, *csiD *(for carbon) and *psiF *(for phosphate) were also expressed higher in EDL933 wild type than in the *rpoS *mutants. Unlike in K12, the genes that encode universal stress proteins *uspA*, *yecG *(*uspC*), *yiiT *(*uspD*), *ydaA *(*uspE*) showed attenuated expression in *rpoS *mutants (this study) while their expression is not dependent on RpoS in K12 [45,46].

#### Transporter and Membrane proteins

**Figure 4:**
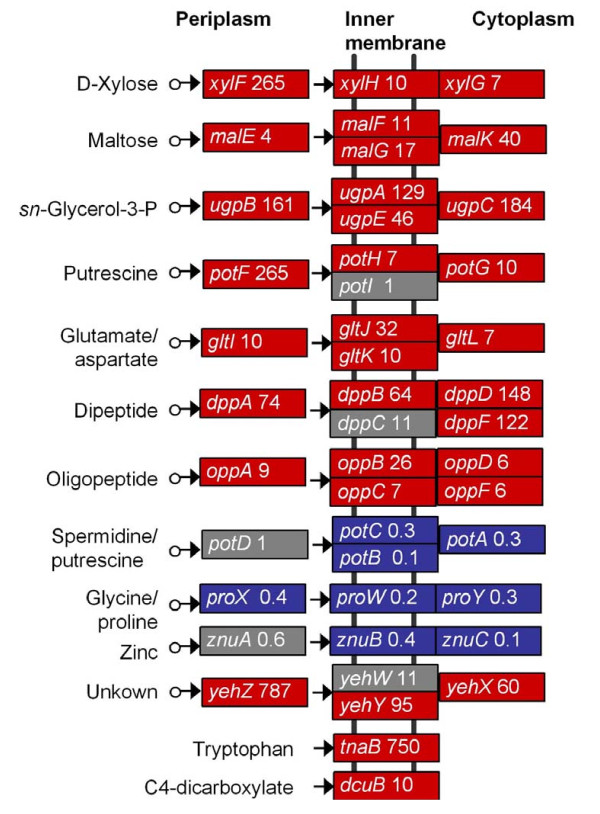
**Effect of RpoS on expression of transporter genes**. The mean expression ratio (MER/RpoS-dependence level) is given after each gene. Genes highlighted in red were expressed higher in wild type, those in blue were expressed higher in the *rpoS *mutant, and those in grey were not found to be significantly different (P > 0.05).

The expression of many genes for nutrient transport was affected by the *rpoS *mutation (Figure 4). Most of these genes encode proteins belonging to the ATP-Binding Cassette (ABC) transporter family. RpoS positively regulated ABC transporter genes included those for transport of oligopeptide (encoded by *oppABCDF*), dipeptide (*dppABDF*), putrescine (*potFGH*), maltose (*malEFGK*), glutamate/aspartate (*gltIJKL*), D-xylose (*xylFHG*) and *sn*-glycerol-3-P (*ugpABCE*). The expression of genes *yehWXYZ*, encoding a predicted ABC transporter, was also highly dependent on RpoS. Transporter genes expressed higher in the *rpoS *mutants included those for spermidine/putrescine (*potABCD*), glycine/proline (*proWXY*), and Zinc (*znuABC*). Besides ABC transporters, the *tnaB *gene encoding a tryptophan transporter and the *dcuB *gene encoding a transporter for C4-dicarboxylates (e.g., fumarate and malate) uptake were expressed at a lower level in the *rpoS *mutants compared with that in wild type EDL933. The gene *cstA*, encoding a peptide transporter that is induced under carbon starvation, has been shown to be negatively regulated by RpoS in a K12 strain [47], while we found that the expression of *cstA *was attenuated in the *rpoS *mutants of EDL933.

#### Metabolism

**Figure 5:**
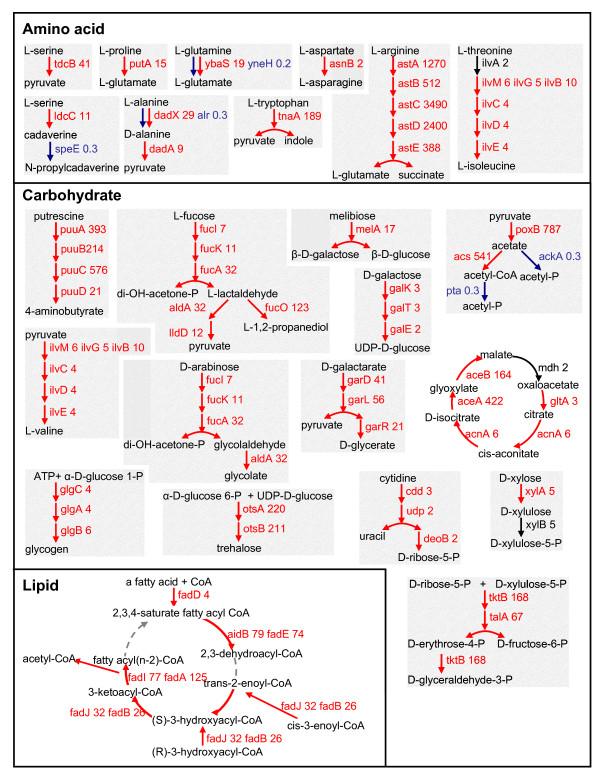
**Metabolic pathways that are regulated by RpoS in stationary phase**. Genes expressed higher in wild type are colored red and those expressed higher in *rpoS *mutants are blue. Genes whose differential expression was not significant (P > 0.05) are in black. The mean expression ratio (MER: WT/*rpoS*) is indicated after each gene.

RpoS had a substantial effect on expression of metabolic genes, primarily for utilization of amino acids and carbohydrates (Figure 5). LB medium is rich in amino acids that can be utilized by *E. coli *as nutrient sources [48]. We found that the expression of genes for utilization of serine (*tdcB*), proline (*putA*), glutamine (*ybaS*), aspartate (*asnB*), arginine (*astCABDE*), tryptophan (*tnaA*), threonine (*ilvBCDEMG*), and alanine (*dadAX*) was expressed higher in the wild type EDL933 than in the *rpoS *mutants. The genes *yneH *and *alr*, encoding isoenzymes of YbaS and DadX, respectively, were expressed higher in the *rpoS *mutants (Figure 5). Pyruvate and glutamate appeared to be two common intermediate metabolites in RpoS-regulated amino acid utilization (Figure 5). For carbohydrate utilization, genes whose expression is positively regulated by RpoS included those encoding for putrescine degradation (*puuABCD*), fatty acid beta-oxidation (*fadBA*, *fadD*, *fadE*, and *fadIJ*), fucose utilization (*fucAO, fucIK, lldD*, and *aldA*), glucarate degradation (*garDLR*), glyoxylate cycle (*aceBA*, *acnA*, and *gltA*), and synthesis of trehalose (*otsBA*) and glycogen (*glgABC*) (Figure 5). The *cdd *and *udp *genes for pyrimidine degradation were reduced in expression in the *rpoS *mutant, while the expression of genes *udk*, *cmk*, *upp*, and *codA *that are involved in the pyrimidine biosynthesis pathway was enhanced.

Some of these metabolic genes may play an important role in colonization and pathogenesis of *E. coli in vivo *in host environments. For example, the expression of *fucAO *is important for colonization of *E. coli *in mouse intestine [49]. Mutants defected in metabolism of maltose and glycogen are also impaired in colonization of EDL933 in mouse intestine [50].

#### Transcription Regulation

The expression of 29 genes encoding known transcriptional regulators was affected by the *rpoS *mutation. Sixteen genes (*lsrR, mhpR, prpR, putA, lldR, hcaR, galS, gadXWE, fucR, dgsA, csgD, cdaR, bolA*, and *xylR*) were expressed higher in the wild type EDL933 while 13 genes (*dicA, deoR, birA, uhpA, marR, metJ, pdhR, purR, rcsA, arsR, asnC, cspA*, and *fis*) were expressed higher in the *rpoS *mutants (Additional file 1). The observed differential expression of many genes in the *rpoS *mutants may be an indirect effect of RpoS through these intermediate regulators. Some regulatory genes are known to be RpoS-controlled, such as *bloA *[51], *gadE *[52], and *csgD *[28]. Expression of the *hcaR *gene, encoding the hydrocinnamic acid regulator, is stationary phase dependent but RpoS-independent in *E. coli *K12 strain [53]. Here we found that expression of *hcaR *was induced in stationary phase in both wild type EDL933 and *rpoS *mutants. However, the induction level was significantly higher in wild type, indicating that RpoS is important for full expression of *hcaR*.

#### Virulence and O-island genes

We found that 10% of the identified RpoS-regulated genes are located on O-islands. Among them, 50 genes were expressed higher in wild type EDL933 in stationary phase (Table 4) while 49 genes expressed higher in the *rpoS *mutants (Table 5). The functions of most of these genes are still unknown. On the LEE island (located on the O-island 148), three genes, *ler*, *cesF *and Z5139, were expressed significantly higher in wild type EDL933 than in the *rpoS *mutants (Table 4), while the *eae *gene, encoding the outer membrane intimin protein essential for colonization and virulence, was expressed twofold higher in *rpoS *mutants (Table 5). The expression of other genes on the LEE islands was not significantly affected by the *rpoS *mutation. The *espI *gene, though not located on the LEE island, encodes a secreted protein whose secretion requires the LEE-encoded type III secretion system [54]. The expression of *espI *was 78 fold higher in the wild type EDL933. The *nlpA *gene, encoding an inner membrane protein that is required for virulence in *Haemophilus influenzae *[55], was impaired in its expression in the *rpoS *mutants. The *dppA *operon, required for colonization by uropathogenic *E. coli *[56], was expressed much higher in the wild type EDL933 than *rpoS *mutants.

**Table 4:** RpoS-dependent EDL933-specific O-island genes (MER ≥ 2, P < 0.05). These are not present in *E. coli *K12 MG1655. MER: mean expression ratio (WT/*rpoS*).

Gene	Expression (log2)		MER	Position	Function
	WT	*rpoS*			
Z0321	12.4 ± 0.0	10.0 ± 0.3	6	O-Island 8	Putative regulator (prophage CP-933H)
Z0443	10.0 ± 0.1	6.7 ± 0.1	10	O-Island 19	Unknown
Z0463	7.2 ± 0.8	2.2 ± 0.0	32	O-Island 20	Putative response regulator
Z0608	10.8 ± 0.4	5.0 ± 1.0	55	O-Island 28	Putative outer membrane export protein
Z0609	6.5 ± 0.6	2.2 ± 0.0	20	O-Island 28	Unknown
Z0701	5.6 ± 0.3	3.7 ± 0.3	4	O-Island 30	Unknown
Z0702	10.4 ± 0.2	9.2 ± 0.1	2	O-Island 30	Unknown (Rhs Element Associated)
Z0957	12.0 ± 0.1	10.6 ± 0.2	3	O-Island 36	Unknown (prophage CP-933K)
Z0958	11.8 ± 0.4	10.0 ± 0.1	3	O-Island 36	Unknown (prophage CP-933K)
Z0984	5.7 ± 0.2	4.2 ± 0.2	3	O-Island 36	Unknown (prophage CP-933K)
Z1129	9.1 ± 0.2	7.9 ± 0.3	2	O-Island 43	Putative enzyme
Z1185	11.5 ± 0.2	10.3 ± 0.2	2	O-Island 43	Unknown
Z1190	12.2 ± 0.7	7.9 ± 0.2	20	O-Island 43	Putative enzyme
Z1193	10.2 ± 0.8	6.3 ± 0.8	15	O-Island 43	Unknown
Z1385	11.8 ± 0.1	10.5 ± 0.3	2	O-Island 44	Unknown (cryptic prophage CP-933M)
Z1386	7.1 ± 0.3	5.8 ± 0.2	2	O-Island 44	Unknown (cryptic prophage CP-933M)
Z1528	6.5 ± 0.3	3.3 ± 0.7	9	O-Island 47	Unknown
Z1629	12.2 ± 0.8	5.3 ± 0.3	117	O-Island 48	Putative enzyme
Z1764	9.0 ± 0.2	7.3 ± 0.2	3	O-Island 50	Putative enzyme (prophage CP-933N)
Z1922	9.9 ± 0.8	4.8 ± 0.2	35	O-Island 52	Unknown (prophage CP-933X)
Z1923	8.9 ± 1.0	2.9 ± 0.1	64	O-Island 52	Unknown (prophage CP-933X)
Z1924	11.1 ± 0.9	4.0 ± 0.2	137	O-Island 52	Unknown (prophage CP-933X)
Z2048	4.1 ± 0.2	2.3 ± 0.1	3	O-Island 57	Unknown (prophage CP-933O)
Z2057	5.9 ± 0.2	4.3 ± 0.4	3	O-Island 57	Putative enzyme (prophage CP-933O)
Z2124	6.0 ± 0.2	5.0 ± 0.1	2	O-Island 57	Unknown (prophage CP-933O)
Z2149	13.4 ± 0.4	10.1 ± 0.3	10	O-Island 57	Unknown (Phage or Prophage Related)
Z2150	10.4 ± 0.6	5.3 ± 0.4	33	O-Island 57	Unknown (Phage or Prophage Related)
Z2151	11.6 ± 0.4	8.6 ± 0.1	8	O-Island 57	Unknown (Phage or Prophage Related)
Z2164	6.8 ± 0.1	4.3 ± 0.6	6	O-Island 59	Putative regulator
Z2254	6.9 ± 0.2	4.7 ± 0.6	5	O-Island 64	Unknown (Rhs Element Associated)
Z2994	8.9 ± 0.2	6.8 ± 0.1	4	O-Island 76	Unknown (prophage CP-933T)
Z3391	9.9 ± 0.5	7.1 ± 0.4	7	O-Island 95	Putative enzyme
Z3392	8.4 ± 0.4	5.0 ± 0.2	11	O-Island 95	Putative enzyme
Z3393	7.4 ± 0.3	2.2 ± 0.0	36	O-Island 95	Putative enzyme
Z3394	6.0 ± 0.1	2.3 ± 0.0	13	O-Island 95	Putative transporter
Z3623	9.4 ± 0.3	4.8 ± 0.1	24	O-Island 102	Sucrose permease
Z3624	8.5 ± 0.2	2.5 ± 0.0	64	O-Island 102	D-fructokinase
Z3625	9.4 ± 0.1	2.2 ± 0.0	139	O-Island 102	Sucrose hydrolase
Z3947	8.3 ± 0.4	4.0 ± 0.5	19	O-Island 108	Unknown (Phage or Prophage Related)
Z4488	7.8 ± 0.2	5.6 ± 0.4	4	O-Island 126	Putative enzyme
Z4803	6.4 ± 0.9	2.4 ± 0.1	17	O-Island 134	Putative enzyme
Z5114	7.4 ± 0.3	4.9 ± 0.4	6	O-Island 148	LEE-encoded virulence protein CesF
Z5139	14.0 ± 0.4	12.0 ± 0.5	4	O-Island 148	LEE-encoded virulence protein
Z5140	14.2 ± 0.3	12.6 ± 0.3	3	O-Island 148	LEE-encoded regulator Ler
Z5199	9.7 ± 0.3	6.6 ± 0.5	8	O-Island 152	Unknown
Z5200	9.0 ± 0.7	3.3 ± 0.2	53	O-Island 152	Unknown
Z5619	7.3 ± 0.3	6.0 ± 0.3	3	O-Island 166	Putative regulator
Z5684	7.3 ± 0.1	3.4 ± 0.5	15	O-Island 167	Putative regulator
Z5887	8.3 ± 0.1	6.2 ± 0.3	4	O-Island 172	Unknown
Z6024	9.3 ± 0.3	3.0 ± 0.1	78	O-Island 71	EspI, essential for virulence

**Table 5:** RpoS negatively regulated genes on the O-islands (P < 0.05). MER: mean expression ratio (*rpoS/*WT).

Gene	Expression (log2)		MER	Position	Function
	WT	*rpoS*			
Z0264	7.8 ± 0.1	9.0 ± 0.0	-2	O-Island 7	Unknown
Z0372	11.4 ± 0.3	12.6 ± 0.2	-2	O-Island 11	Unknown
Z0397	5.1 ± 0.3	6.2 ± 0.1	-2	O-Island 14	Unknown
Z0955	9.7 ± 0.3	11.5 ± 0.0	-4	O-Island 36	Unknown (prophage CP-933K)
Z1146	11.7 ± 0.3	12.7 ± 0.3	-2	O-Island 43	Putative urease accessory protein E
Z1144	11.3 ± 0.2	12.4 ± 0.2	-2	O-Island 43	Putative urease structural subunit B
Z1142	10.9 ± 0.3	12.1 ± 0.2	-2	O-Island 43	Putative urease accessory protein D
Z1164	12.1 ± 0.1	13.4 ± 0.0	-2	O-Island 43	Unknown
Z1143	10.9 ± 0.3	12.3 ± 0.2	-3	O-Island 43	Putative urease structural subunit A
Z1160	3.7 ± 0.1	5.5 ± 0.4	-4	O-Island 43	Unknown
Z1163	7.5 ± 0.5	9.4 ± 0.4	-4	O-Island 43	Unknown
Z1346	11.9 ± 0.1	13.0 ± 0.2	-2	O-Island 44	Unknown (cryptic prophage CP-933M)
Z1348	10.8 ± 0.1	11.9 ± 0.2	-2	O-Island 44	Unknown (cryptic prophage CP-933M)
Z1324	4.4 ± 0.1	5.8 ± 0.3	-3	O-Island 44	Putative exoDNaseVIII
Z1347	10.0 ± 0.0	11.5 ± 0.2	-3	O-Island 44	Unknown (cryptic prophage CP-933M)
Z1326	3.4 ± 0.3	5.5 ± 0.3	-4	O-Island 44	Putative inhibitor of cell division
Z1325	4.1 ± 0.4	6.3 ± 0.3	-5	O-Island 44	Unknown (cryptic prophage CP-933M)
Z1456	12.8 ± 0.2	13.8 ± 0.3	-2	O-Island 45	Unknown (bacteriophage BP-933W)
Z1503	8.0 ± 0.5	10.2 ± 0.5	-4	O-Island 45	Unknown (bacteriophage BP-933W)
Z1794	5.6 ± 0.3	6.8 ± 0.3	-2	O-Island 50	Putative holin protein
Z1878	13.0 ± 0.2	14.7 ± 0.1	-3	O-Island 52	Putative Bor protein
Z2146	5.8 ± 0.2	7.0 ± 0.1	-2	O-Island 57	Putative OMP Lom precursor
Z2100	2.4 ± 0.1	3.7 ± 0.2	-2	O-Island 57	Unknown (prophage CP-933O)
Z2045	9.9 ± 0.1	11.4 ± 0.1	-3	O-Island 57	Regulator of DicB
Z2105	8.8 ± 0.2	10.3 ± 0.1	-3	O-Island 57	Unknown (prophage CP-933O)
Z2101	3.8 ± 0.0	5.3 ± 0.3	-3	O-Island 57	Putative endonuclease
Z2103	10.5 ± 0.1	12.0 ± 0.1	-3	O-Island 57	Unknown (prophage CP-933O)
Z2144	5.9 ± 0.2	7.6 ± 0.2	-3	O-Island 57	Putative tail component of CP-933O
Z2059	5.3 ± 0.3	8.7 ± 0.3	-11	O-Island 57	Unknown (prophage CP-933O)
Z2510	5.0 ± 0.4	7.0 ± 0.2	-4	O-Island 70	Putative transcriptional repressor
Z3201	12.0 ± 0.3	13.2 ± 0.2	-2	O-Island 84	O antigen flippase Wzx
Z3361	7.3 ± 0.2	8.3 ± 0.1	-2	O-Island 93	Putative regulatory protein
Z3360	11.8 ± 0.1	13.0 ± 0.2	-2	O-Island 93	Unknown (prophage CP-933V)
Z3322	5.0 ± 0.2	6.3 ± 0.2	-2	O-Island 93	Putative major tail subunit
Z3622	6.9 ± 0.2	10.1 ± 0.7	-9	O-Island 102	Putative resolvase
Z4048	8.4 ± 0.2	10.4 ± 0.1	-4	O-Island 110	Putative regulator
Z4789	3.1 ± 0.2	4.4 ± 0.1	-2	O-Island 133	Unknown
Z4851	7.4 ± 0.0	8.6 ± 0.2	-2	O-Island 138	Unknown
Z4855	9.4 ± 0.2	10.5 ± 0.1	-2	O-Island 138	Unknown
Z4852	8.9 ± 0.2	10.1 ± 0.1	-2	O-Island 138	Putative acyltransferase
Z4857	3.5 ± 0.3	4.9 ± 0.3	-3	O-Island 138	Unknown
Z4854	8.7 ± 0.3	10.2 ± 0.1	-3	O-Island 138	Putative acyl carrier protein
Z4861	3.2 ± 0.5	5.7 ± 0.4	-6	O-Island 138	Unknown
Z4860	6.3 ± 0.3	8.8 ± 0.2	-6	O-Island 138	Unknown
Z5051	10.2 ± 0.3	11.4 ± 0.1	-2	O-Island 145	Putative LPS biosynthesis enzyme
Z5049	11.7 ± 0.3	13.5 ± 0.3	-3	O-Island 145	Putative LPS biosynthesis enzyme
Z5089	3.8 ± 0.2	4.9 ± 0.1	-2	O-Island 148	Putative transposase
Z5110	7.6 ± 0.2	8.9 ± 0.1	-2	O-Island 148	LEE-encoded virulence protein Eae
Z5225	3.6 ± 0.2	4.7 ± 0.2	-2	O-Island 154	Putative major fimbrial subunit

- Indicates negative regulation.

### Western blot analysis of LEE proteins under LEE-induction conditions

**Figure 6:**
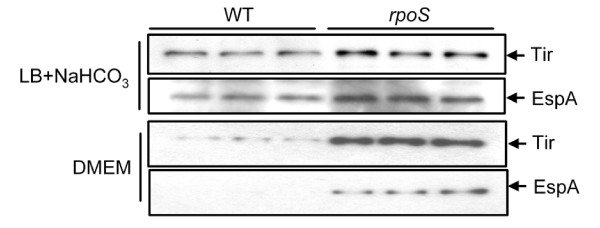
**Western blot analysis of Tir and EspA expression in wild type and *rpoS *mutants**. Cultures were grown aerobically at 37°C in LB media supplemented with 44 mM NaHCO_3 _to OD_600 _= 1.5 or in DMEM media in 5% CO_2 _(two known LEE-induction conditions). Cell pellets were resuspended in SDS loading buffer and boiled for 5 min. Resultant cell extracts were resolved on a 10% SDS-PAGE gel. Proteins were transferred to a PVDF membrane by electrophoresis, followed by incubation of the membrane with anti-Tir or anti-EspA specific antibody. Signals were detected using ECL solution and Hyperfilm-ECL film (Amersham).

Growth condition plays a considerable effect on LEE gene expression [57,58]. The expression of LEE genes is low in LB media and is induced in LB supplemented with sodium bicarbonate or DMEM media in 5% CO_2 _[57,58]. To determine whether the expression of LEE genes was controlled by RpoS under these LEE-induction conditions, we examined the expression of one gene from each of the five LEE islands by qPCR using cultures grown in LB supplemented with 44 mM sodium bicarbonate media [57]. All genes tested were expressed higher in the *rpoS *mutants. The ratio of expression in *rpoS *mutants verse wild type EDL933 for *ler *(LEE1), *sepZ *(LEE2), *escV *(LEE3), *tir *(LEE4), *sepL *(LEE5), *grlR *and *grlA *(LEE regulator) was 2.8 ± 0.5, 1.3 ± 0.4, 5.5 ± 0.4, 4.8 ± 0.4, 6.4 ± 0.4, 4.7 ± 0.4, and 7.6 ± 0.4, respectively. Western blot analysis revealed that the expression of Tir and EspA was enhanced in the *rpoS *mutants of EDL933 (Figure 6). Similar results were obtained in cultures grown in DMEM media, another LEE induction condition (Figure 6). Consistent with previous results, neither Tir nor EspA was detected in LB without sodium bicarbonate (data not shown).

### Negative regulation by RpoS

**Figure 7:**
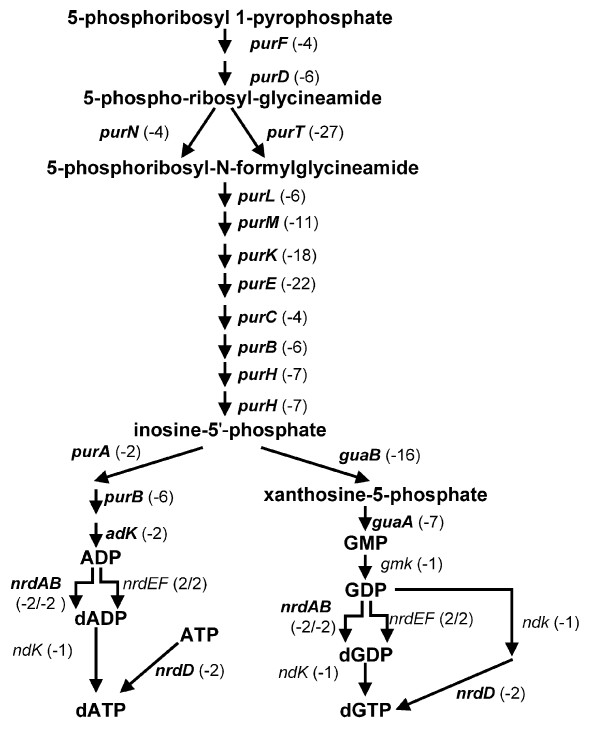
**RpoS-regulation of genes required for *de novo *biosynthesis of purine nucleotides pathway I in stationary phase**. RpoS-dependence (MER) is indicated in parentheses. A negative value (-) denotes RpoS-negative regulation. The pathway map is adapted from the EcoCyc database. Genes that were significantly differentially expressed (P < 0.05) are highlighted in bold.

**Figure 8:**
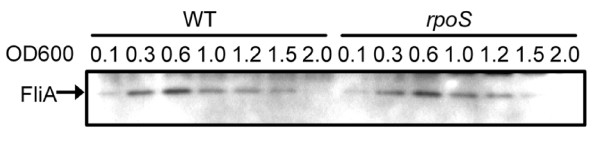
**Expression of FliA in WT and *rpoS *mutants of EDL933 in LB**. Western blot analyses of the expression of the flagella sigma factor FliA were performed using monoclonal antibody to FliA as described in Material and Methods. To confirm equal protein loading, another protein gel run in parallel was stained by Coomassie blue R250.

As mentioned above, we found 536 genes expressed higher in *rpoS *mutants in stationary phase (Table 3 and Additional file 1). These genes are involved in many cellular functions, including metabolism (e.g., *thiI *and *guaBA*), nutrient transport (e.g., *ampG*, *cmr *and *uraA*), and DNA modification (e.g., *endA *and *nth*). The expression of almost all genes in the purine biosynthesis pathway was enhanced in the *rpoS *mutant (Figure 7). The *rsxABCDGE *operon that is required for the reduction of SoxR was also expressed higher in the *rpoS *mutants (Table 3). Interestingly, the flagellar genes and the TCA cycle genes, whose expression is negatively regulated by RpoS in *E. coli *K12 strains [15], were not differentially expressed in the *rpoS *mutant of EDL933. The flagellar sigma factor FliA, was expressed similarly in wild type EDL933 and *rpoS *mutants (Figure 8).

### Verification of microarray results

**Figure 9:**
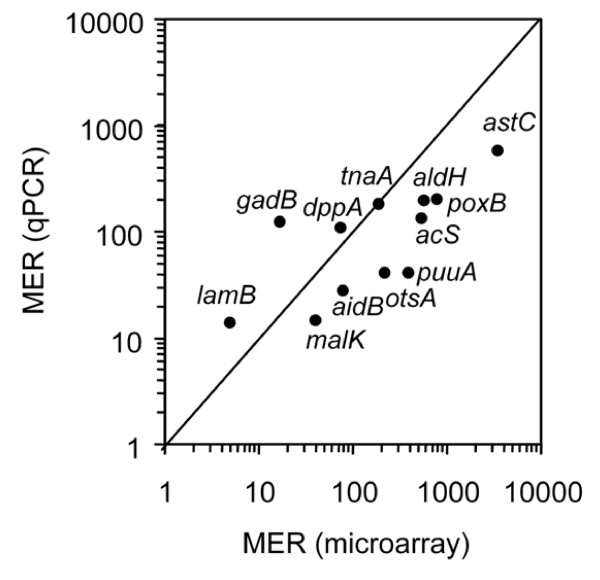
**Confirmation of microarray data using qPCR**. RpoS dependence is represented by the mean expression ratio (WT/*rpoS*).

To validate the microarray results, we determined the expression level and RpoS dependence of candidate genes by qPCR (Figure 9). The RpoS-dependence levels of all 12 genes tested were in good correlation between results of microarray and qPCR. Because the *rpoS *sequence is absent in the *rpoS *null mutant tested in this study, the signal difference for *rpoS *between wild type EDL933 and *rpoS *mutant strains serves as an internal control for the sensitivity of microarray data. We found the expression difference of the two *rpoS *probe sets was about 5,000 fold between wild type and *rpoS *mutants. As expected, we also found many known RpoS-regulated genes (e.g., *osmY*, *katE *and *astC*) were identified as RpoS-controlled genes in this study.

## Discussion

In this study, we have characterized the RpoS regulon of the important pathogenic *E. coli *O157:H7 strain EDL933. Comparison with previous data obtained using laboratory K12 strains reveals substantial differences between the composition of RpoS regulon in K12 and O157:H7 EDL933. As might be expected, the RpoS-regulon identified in EDL933 is much larger than that of K12, which is partly attributable to the larger number of genes present in the pathogenic strain. Another factor may be different levels of the expression of RpoS itself. Indeed, we found that the level of RpoS was higher in EDL933 than in MG1655 in early stationary phase (Additional file 2), consistent with previous results that RpoS levels vary among *E. coli *isolates [59]. Though there is a core set of genes regulated by RpoS in both K12 and EDL933 strains, the RpoS-dependence of a large number of genes (~80% of RpoS-dependent genes in EDL933) is strain-specific, including a group of RpoS-dependent genes on O-islands and several virulence determinant genes. RpoS has a larger effect on exponential phase gene expression in K12 strain than in EDL933 [18,19]. These results suggest that RpoS regulation may be strongly dependent on strain background. Consistent with this, there are many known phenotypic differences between K12 and EDL933. For example, MG1655 and EDL933 differ in utilization of nutrients and location of colonization during *in vivo *growth in mouse intestine [50,60,61].

The expression of a large number of genes was higher in the *rpoS *mutants, indicating negative control of RpoS on gene expression. As a sigma factor, negative control exerted by RpoS is likely an indirect effect, probably resulting from sigma factor competition [45]. Because the number of sigma factors exceeds that of core RNA polymerase, different sigma factors compete for binding to the core enzyme [62]. Deletion of RpoS, a major sigma factor in stationary phase, may thus result in increased amount of core enzyme associated with other sigma factors and their-directed gene expression. In *E. coli *K12 strain, there is also a large number of genes negatively regulated by RpoS [15]. For example, expression of genes for chemotaxis and flagella is negatively regulated by RpoS in K12 [15,17]. However, this was not the case in EDL933 (this study), suggesting the negative regulation of RpoS was also strain-specific. In other pathogens, the effect of RpoS on flagella expression is variable (Table 6) [15,17,63-71]. In *P. aeruginosa*, expression of the flagellar gene *fliF *as well as genes for chemotaxis is positively regulated by RpoS [64]. In *Vibrio cholerae*, RpoS positively controls the expression of chemotaxis and flagellar genes during pathogenesis [68]. In *Legionella pneumophila *and *S. typhimurium*, RpoS is important for expression of flagella [63,65]. However, flagella gene expression is independent of RpoS in *S. typhimurium *strain LT2 [66], which has a mutant allele of RpoS [72].

**Table 6:** Effect of RpoS on expression of flagella and chemotaxis genes.

Species	Flagella or Motility	Chemotaxis	Reference
*E. coli *K12	Down	Down	[15,17,70,71]
*E. coli *O157:H7	-^a^	-	This study
*Legionella pneumophila*	Up	ND^b^	[63]
*Pseudomonas aeruginosa*	Up	Up	[64]
*Salmonella enteritidis*	Up	ND	[65]
*S. typhimurium *LT2	-	ND	[66]
*S. typhimurium *SL1344	Up	ND	[67]
*Vibrio cholerae*	Up	Up	[68]
*Vibrio vulnificus*	UP	ND	[69]

^a ^Indicates no effect.

^b ^Not determined.

The intestinal growth environment inhabited by EHEC *E. coli *is complex. Utilization of glycogen [50], maltose [50], L-fucose [49], galactose [61], arabinose [61], and ribose [61] is important for colonization by *E. coli*. We found that an *rpoS *mutation attenuates the expression of genes involved in metabolism of these sugars (Figure 5), suggesting a role of RpoS in regulation of bacterial colonization. This is consistent with our previous findings in an animal model that wild type *C. rodentium *colonizes mouse colon better than *rpoS *mutants [26]. The contribution of RpoS-regulated metabolism to *in vivo *colonization needs to be further evaluated through construction of mutations in relevant pathways to identify specific causal factors.

The expression of most genes on the LEE island is under control of Ler, a LEE-encoded regulator [73,74], and thus LEE genes is expected to be expressed similarly. However, previous results have shown that this is not the case [75,76]. Consistent with this, our results show that RpoS had an opposing effect on LEE gene expression, suggesting that LEE genes are under differential control for expression. The difference in expression of LEE genes may be due to the lack of induction signals for LEE expression in LB. Under induction conditions, all LEE genes tested were expressed higher in the *rpoS *mutants (this study).

A recent microarray study reviewed differences in the heat shock response of *E. coli *O157:H7 EDL933 and K12 strains, and attributed discrepancies to experimental conditions and/or genomic compositions [77]. About 30 EDL933 specific genes are differentially expressed during heat shock [77]. Only four of the top 25 heat shock response genes were RpoS-dependent (this study), suggesting that other regulators (e.g., the heat shock sigma factor RpoH) are required for the full heat shock response. Again, differences in methodology (e.g., array platforms and experimental conditions) make it difficult to directly compare results.

Gene expression profiling has greatly improved our knowledge of the role of RpoS in regulation of genes and many cellular functions. However, we are still far from fully understanding the physiological role of RpoS. For example, a large portion of RpoS-regulated genes are those with unknown or putative functions. Factors responsible for strain-specific effects also remain elusive. Furthermore, the regulation of RpoS itself is not fully understood. Recent studies have identified two anti-adaptor proteins, IraM (previously known as YcgW) [78] and IraD (YjiD) [20], which stabilize RpoS through inhibition of RssB-ClpXP directed proteolysis. RpoS activity has also been found to be transiently inhibited by FliZ in post exponential phase [79]. It is likely that there are other unidentified factors involved in the regulatory network of RpoS.

## Conclusion

Our results reveal the first snapshot overview of RpoS-regulated transcriptome expression in non-K12 strains. This, together with previous results regarding RpoS control in laboratory strains, provides a useful database for understanding how global regulators (e.g., RpoS) can gain additional functions in pathogenic *E. coli *strains.

## Methods

### Strains, media and growth conditions

*E. coli *strain O157:H7 EDL933 and its *rpoS *mutant derivative were employed in this study. Cultures were grown aerobically at 37°C with shaking at 200 rpm in Luria-Bertani media, and growth was monitored spectrophotometrically at OD_600_. Antibiotics were used at the following concentrations: ampicillin (100 μg/ml) and chloramphenicol (25 μg/ml).

### Construction of EDL933 *rpoS *deletion mutant

An *rpoS *non-polar deletion mutant was constructed by homologous recombination as described previously [80,81]. Briefly, a linear DNA fragment, harboring the chloramphenicol resistant gene *cat *and homologous *rpoS*-flanking sequences, was amplified using pKD3 plasmid (template) and primers FP1 (CCTCGCTTGAGACTGGCCTTTCTGACAGTGCTTACGTGTAGGCTGGAGCTGCTTC) and RP1 (ATGTTCCGTCAAGGGATCACGGGTAGGAGCCACCTTCATATGAATATCCT CCTTAG) and introduced into EDL933 competent cells by electroporation. Transformants were selected on LB chloramphenicol plates. The *cat *gene was further removed by recombination with the FLP recombinase. The loss of *rpoS *was confirmed by PCR using flanking primers and by sequencing.

### RNA preparation

RNA samples were prepared as previously described [18]. Overnight cultures were diluted into fresh media at a starting OD_600 _of 0.0001 to allow cells to grow at least ten generations prior to RNA isolation in exponential phase. Cultures grown in triplicate were sampled at OD_600 _= 0.3 (exponential phase) and OD_600 _= 1.5 (stationary phase), conditions used in our previous studies for comparison [15,18]. RNA samples were prepared using hot acidic phenol (pH 4.3, Sigma-Aldrich), and the quality of RNA was examined using a Bioanalyzer 2100 (Agilent Technologies).

### Microarray analysis

The Affymetrix GeneChip *E. coli *Genome 2.0 Array was employed in this study. This array chip contains more than 10,000 probe sets that cover all genes in the genomes of four type *E. coli *strains, K12 MG1655, O157:H7 EDL933, O157:H7 Sakai, and the uropathogen, CFT073. A gene that is present in all genomes with high similarity in sequence is represented by a single probe set. Although this is an effective approach to minimize the total number of probe sets used to cover all four genomes, some homologous genes with low sequence similarity in the four strains may be represented by more than one probe set. For example, there are two probe sets in the array representing *rpoS *(probe set IDs: 1761030_s_at and 1767783_s_at) because the *rpoS *sequence in the strain CFT073 harbors an internal mutation that results in two truncated genes, c3306 (519 bp probing to 3' end of *rpoS*) and c3307 (435 bp probing to 5' end of *rpoS*). Both probe sets hybridized to *rpoS *transcripts and the resultant signals in wild type samples were 4,939 and 7,643 time higher than those in the knockout *rpoS *mutants, respectively (this study). Though both probe sets are representative of *rpoS*, this leads to duplication. To avoid this problem, microarray data were curated to remove redundant probe sets in our analysis. Microarray samples were analyzed using dChip [82] and BRB Arraytools [83], as described previously [17]. Samples were log_2 _transformed and normalized using the GCRMA method [84]. RpoS dependence of genes is represented by the mean expression ratio (MER) of WT and *rpoS *mutants. The significance of expression difference was tested using Student's t-tests. Genes with MER value ≥2 or ≤0.5 and P value < 0.05 were considered to be controlled by RpoS [17]. The false discovery rate (FDR) was estimated by 1,000 time random permutations as previously described [17]. Microarray data can be accessed in the Gene Expression Omnibus database at the National Center for Biotechnology Information under the accession number GSE17420.

### Quantitative real-time PCR (qPCR)

To confirm microarray results, we tested gene transcription by qPCR as previously described [17]. Primers were designed using the PerlPrimer program [85] and synthesized by the MOBIX laboratory at McMaster University. RNA samples were prepared as for microarray analysis. First strand cDNA was synthesized using a cDNA synthesis kit (New England Biolabs). Gene amplification was detected using SYBR green (Clontech) in a MX3000P qPCR system (Stratagene). The expression level of genes was determined by constructing a standard curve using serial dilutions of EDL933 genome DNA with known concentrations. The 16S RNA gene, *rrsA*, was used as a reference control to normalize differences in total RNA quantity among samples [86].

### Western blot analyses

Cultures were grown in LB media aerobically at 37°C and sampled periodically. Samples were immediately mixed with chloramphenicol (150 μg/ml) and placed on ice to stop protein synthesis, followed by centrifugation at 15,000 × g for 2 min. Cell pellets were flash frozen in liquid nitrogen prior to use. Cell pellets were thawed on ice, resuspended to OD_600 _= 1.0 with SDS loading buffer, and boiled for 5 min. Samples of 10 μl were resolved on 10% SDS-PAGE and transferred to PVDF membrane [17]. The PVDF membrane was then blocked with 5% milk solution, incubated with mouse monoclonal antibodies for RpoS (NeoClone, Madison, WI), Tir or EspA (a gift from B. Coombes), and HRP-conjugated Goat anti-mouse secondary antibody (Bio-Rad, Hercules, CA). The signal was detected using the ECL solution (Amersham, Pittsburgh, PA) and Hyperfilm-ECL film (Amersham, Pittsburgh, PA). To ensure that equal amounts of protein were loaded, another SDS-PAGE gel was run in parallel and stained with Coomassie Blue R-250.

### Survival of mutants upon exposure to stress conditions

Stationary phase cultures were washed and diluted in 0.9% NaCl before exposure to stress. A total number of 1.0 × 10^8 ^cells were exposed to 1 ml of acidic LB (pH2.5, adjusted with HCl) and 15 mM H_2_O_2_, respectively, while 5.0 × 10^3 ^cells were treated at 55°C for heat exposure. Viable cells were enumerated by serial plating on LB media, and survival expressed as a percentage determined by dividing the number of viable cells by the number of cells before treatment.

## Authors' contributions

TD performed the experiments and wrote the manuscript. HES is the principal investigator who supervised the project and revised the manuscript. Both authors read and approved the final manuscript.

## Supplementary Material

Additional file 1 **Expression of RpoS-regulated genes**. The data show the expression of RpoS-regulated genes (twofold, P < 0.05) in *E. coli *O157:H7 EDL933 wild type and *rpoS *mutants in LB stationary phase (OD_600 _= 1.5) Click here for file

Additional file 2 **Western blot analysis of RpoS expression in *Escherichia coli *O157:H7 strain EDL933 and *E. coli *K12 strain MG1655**. Cultures of EDL933 and MG1655 were grown in LB media at 37°C with vigorous shaking at 200 rpm and sampled at OD_600 _= 0.3 in exponential phase, OD_600 _= 1.5 in stationary phase, and overnight. The *rpoS *mutant of EDL933 was included as a negative control (lane 7). The beta subunit of RNA polymerase RpoB serves as an internal loading control. The expression level of RpoS was higher in EDL933 than in MG1655 in exponential (OD_600 _= 0.3) and early stationary phase (OD_600 _= 1.5). However, the level of RpoS reached a higher level in MG1655 than EDL933 in overnight samples. This is consistent with previous reports that RpoS expression varies depending on strain background (King et al., 2004; See the manuscript for reference). Click here for file
