# Supplementary material for: Global effect of RpoS on gene expression in pathogenic Escherichia coli O157:H7 strain EDL933
Source: BMC Genomics. 2009 Aug 3;10:349. doi: 10.1186/1471-2164-10-349 (PMC2907692; doi:10.1186/1471-2164-10-349)

Additional file 2. **Western blot analysis of RpoS expression in *Escherichia coli* O157:H7 strain EDL933 and *E. coli* K12 strain MG1655.** Cultures of EDL933 and MG1655 were grown in LB media at 37°C with vigorous shaking at 200 rpm and sampled at OD_{600}=0.3 in exponential phase, OD_{600}=1.5 in stationary phase, and overnight. The *rpoS* mutant of EDL933 was included as a negative control (lane 7). The beta subunit of RNA polymerase RpoB serves as an internal loading control. The expression level of RpoS was higher in EDL933 than in MG1655 in exponential (OD_{600}=0.3) and early stationary phase (OD_{600}=1.5). However, the level of RpoS reached a higher level in MG1655 than EDL933 in overnight samples. This is consistent with previous reports that RpoS expression varies depending on strain background (King et al., 2004; See the manuscript for reference).

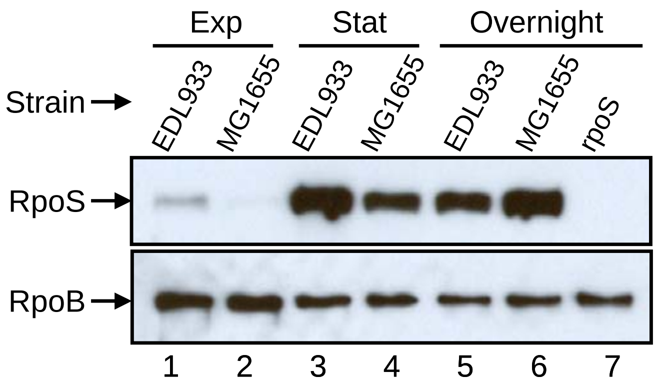

Supplement: Additional file 2 — Western blot analysis of RpoS expression in Escherichia coli O157:H7 strain EDL933 and E. coli K12 strain MG1655. Cultures of EDL933 and MG1655 were grown in LB media at 37°C with vigorous shaking at 200 rpm and sampled at OD600 = 0.3 in exponential phase, OD600 = 1.5 in stationary phase, and overnight. The rpoS mutant of EDL933 was included as a negative control (lane 7). The beta subunit of RNA polymerase RpoB serves as an internal loading control. The expression level of RpoS was higher in EDL933 than in MG1655 in exponential (OD600 = 0.3) and early stationary phase (OD600 = 1.5). However, the level of RpoS reached a higher level in MG1655 than EDL933 in overnight samples. This is consistent with previous reports that RpoS expression varies depending on strain background (King et al., 2004; See the manuscript for reference). [file 1471-2164-10-349-S2.pdf]
